# Surrogate indicators of sensitivity in gynecologic cytology: Can they be used to improve the measurement of sensitivity in the laboratory?

**DOI:** 10.4103/1742-6413.56359

**Published:** 2009-10-09

**Authors:** Andrew A Renshaw, Fadi Brimo, Manon Auger

**Affiliations:** 1 Comprehensive Pathology Associates, Miami, FL, USA; 2 Department of Pathology, McGill University Health Center, McGill University, Montreal, QC, Canada

**Keywords:** Diagnostic accuracy, gynecological cytology, improvement, performance, quality control, rapid pre-screening, routine screening, sensitivity

## Abstract

**Background:**

Measuring the sensitivity of screening in gynecologic cytology in real life is problematic. However, other quality measures may correlate with sensitivity, including the atypical squamous cells (ASC)/squamous intraepithelial lesion (SIL) ratio. Whether these other measures can function as “surrogate indicators” for sensitivity and improve the assessment of sensitivity in the laboratory is not known.

**Materials and Methods:**

We compared multiple quality measures with true screening sensitivity in a variety of situations.

**Results:**

The abnormal rate, ASC rate, and ASC/SIL ratio were all highly correlated (r =.83 or greater) with sensitivity when the overall laboratory sensitivity was low (85%) but became less correlated (.64 or less) or uncorrelated when the screening sensitivity was higher (88% or 95%, respectively). Sensitivity was more highly correlated with the abnormal rate than the ASC/SIL ratio at low screening sensitivity. While thresholds could be set that were highly sensitive and specific for suboptimal screening, these thresholds were often less than one standard deviation away from the mean.

**Conclusion:**

The correlation of the abnormal rate and the ASC/SIL ratio with sensitivity depends on overall sensitivity. Standards to define minimum screening sensitivity can be defined, but these standards are relatively narrow. These features may limit the utility of these quality measures as surrogates for sensitivity.

## INTRODUCTION

Screening sensitivity is critical in gynecologic cytology but difficult to perform in the real life laboratory situation. Although most laboratories in the USA measure the false negative proportion,[1] this has been shown to be inaccurate and to routinely overestimate the true sensitivity of screening.[2] For example, while most laboratories report sensitivities well above 95% using rescreening, large blinded cross-over studies have consistently shown that the sensitivity of screening of routine Pap smears is around 80%.[3] The major limitation with this method is the insensitivity of the rescreen, which has been shown to be only 30% at a threshold of atypical squamous cells (ASC) and 0% at higher thresholds.[2] Because there are no controls when negative slides are rescreened, there is no way to account for this error.

An alternative approach is to use prescreening instead of rescreening. This method allows the sensitivity of the prescreen to be measured and accounted for, by using the abnormal cases that are prescreened as controls. Measurements of sensitivity have been obtained using this method that are much closer to those shown in large blinded cross-over studies, and are presumably more accurate as a result.[4–13] Nevertheless, this method is not routinely used in the USA, though it is practiced in both Canada and the United Kingdom.

As a result, most laboratory directors in the USA have only limited information about the sensitivity of screening in their laboratory. Any method that could improve the evaluation of the cytotechnologists (CTs) in their laboratory may be of value. Recently, it has been shown that other quality measures, such as the atypical squamous cells/squamous intraepithelial lesion (ASC/SIL) ratio may correlate with sensitivity.[12] Whether the ASC/SIL ratio correlates with sensitivity in other laboratory settings, or whether other quality measures may also correlate with sensitivity and serve as a surrogate indicator for sensitivity is not known. To further assess this, we correlated multiple quality measures with multiple screening sensitivities within a laboratory.

## MATERIALS AND METHODS

Rapid prescreening (RPS) for a period of 16 consecutive months was performed as previously described[7] and used to determine the screening sensitivity of individual CTs and the laboratory as a whole. In brief, from November 2006 to February 2008, RPS was routinely performed by up to 15 different CTs depending on the time period on all routine conventional Pap smears (*n* = 51,792) received at the Cytopathology Laboratory of the McGill University Health Center. Because the usual practice in our laboratory is that all Pap smears from high-risk cases, such as those from the colposcopy and oncology clinics, never undergo RPS and are instead always reviewed by a pathologist even if screened as “negative,” all such cases were excluded from the current study. In other words, all the cases included in the current study relate to a routine screening population. The cases included in the study underwent RPS in a manner similar to that which we have previously reported[8,9] with the following modifications. The majority of screeners spend between 15 and 30 min to rapidly prescreen one set of approximately 20 slides each day, allowing 45–90 s to screen each slide. One half of the screeners use the turret method while the others use either the whole or the step method, depending on their preference. The great majority of screeners did not perform the RPS first thing in the morning; the period of the day devoted to RPS is variable from one screener to another. The current study evaluates real life RPS performance done without restriction.

All RPS diagnoses were recorded as abnormal/review (R) or negative (N) on a standardized worksheet, without making any marks on the slide or paperwork. The threshold for (R) was ASC. After the cases were rapidly prescreened, they were fully screened without knowledge of the RPS diagnosis, making sure that the full screener was not the same as the RPS screener. Once a diagnosis was made on full screening (FS), the final and RPS diagnoses were compared. In cases where both reviews were labeled N, the results were finalized by the CT. Cases labeled “R” by both screeners or “N” by RPS but “R” by FS were referred to the pathologist for final diagnosis. Cases labeled “R” by RPS but “N” by FS were referred back to the rapid prescreener to review the slide and dot suspected abnormal cells; these were also referred to a pathologist. The final diagnosis of the pathologists was used as the “gold standard” for calculating sensitivity and specificity of RPS and FS. Four pathologists diagnosed all the cases during the study period; all four had subspecialty training in cytopathology.

Sensitivity of routine screening was calculated for individual CTs and for the laboratory overall in each of the two 8-month study periods. A comparison of individual performances in these two periods was undertaken. Of note is that when calculating the sensitivity of routine screening, we used the more appropriate “corrected” rather than the uncorrected value as described by Renshaw and used in our previous studies.[7,14,15] In brief, the sensitivity of routine screening was considered to be overestimated by the sensitivity of RPS and was recalculated by obtaining a correction factor (CF). The CF was calculated by dividing 100 (%) by the sensitivity of RPS. The FS false-negative rate (FNR) was multiplied by this CF and the result was considered to be the real FNR of routine screening. This value was consequently used to obtain the corrected sensitivity; sensitivity = 1 – FNR. The same laboratory correction factor was used to calculate the true sensitivity of each CT.

The ASC rate and ASC/SIL ratio were calculated from the laboratory data. The total abnormal rate included all diagnoses other than “Negative for Intraepithelial Lesion or Malignancy” (NILM) and Unsatisfactory. The sensitivities we report are at a threshold of ASC. There were insufficient data to measure the sensitivity at thresholds of Low-grade Squamous Intraepithelial Lesion (LSIL) or High-grade Squamous Intraepithelial lesion (HSIL).

Correlations were performed using a Pearson correlation between the variables of interest.

## RESULTS

A total of 51,792 cases were rapidly prescreened. The number of cases reviewed by individual technologists ranged from 281 to 6798. The sensitivity of the laboratory varied from 85% to 88% for the two time periods. Individual CTs had sensitivities from 64% to 100%.

The correlation and variation of a variety of quality measures are summarized in Tables 1–3. Each table represents the laboratory with a different overall sensitivity (85%, 88%, and 95%). Table 1 is from the first 8 months, and includes 11 individual CTs. Table 2 is from from the second time period, and includes 15 individual CTs. Table 3 represents a subset of CTs from the second period (11 CTs) selected to achieve a laboratory sensitivity of 95%.

**Table 1:** Linear correlation of surrogate indicators and sensitivity (with lab sensitivity = 85%; no. of cytotechnologists = 11)

	*Mean*	*SD*	*Range*	*Correlation*
Abnormal rate (%)	3.1	0.8	2.0–4.5	.83
ASC rate only	2.1	0.8	0.9–2.9	.84
ASC and ASC-H rate only	2.3	0.9	0.9–3.3	.85
ASC/SIL	2.3	1.4	0.9–4.5	.67

**Table 2:** Linear correlation of surrogate indicators and sensitivity (with lab sensitivity = 88%; no. of cytotechnologists = 15)

	*Mean*	*SD*	*Range*	*Correlation*
Abnormal rate (%)	3.1	0.8	1.9–6.0	.28
ASC rate only	1.8	0.8	0.8–3.9	.54
ASC and ASC-H rate only	2.0	0.9	1.0–3.9	.50
ASC/SIL	1.7	1.0	0.6–3.2	.64

**Table 3:** Linear correlation of surrogate indicators and sensitivity (with lab sensitivity = 95%; no. of cytotechnologists = 11)

	*Mean*	*SD*	*Range*	*Correlation*
Abnormal rate (%)	3.3	1.1	1.9-6.0	–.03
ASC rate only	2.0	1.0	0.8–3.9	–.02
ASC and ASC-H rate only	2.2	1.0	1.0–3.9	–.04
ASC/SIL	1.9	1.0	0.6–3.2	–.01

The abnormal rate, ASC rate, and ASC/SIL ratio were all highly correlated (*r* =.83 or greater) with sensitivity when the overall laboratory sensitivity was low (85%) but became less correlated (.64 or less) or uncorrelated when the screening sensitivity was higher (88% or 95%, respectively). Sensitivity was more highly correlated with the abnormal rate than ASC in laboratories with lower screening sensitivity. The inclusion of Atypical Squamous cells; cannot rule out HSIL (ASC-H) with ASC made only a marginal difference.

Thresholds could be defined as that which were useful in identifying individual CTs whose screening sensitivity was suboptimal [Tables 4 and 5], though in most cases the thresholds were less than one standard deviation below the mean. For example, in Table 4 if one wished to ensure that the screening sensitivity of individual CTs was at least 85%, then one would need to be sure that the total abnormal rate for each CT was at least 2.3%. If one wished to ensure that the screening sensitivity of individual CTs was at least 90%, one would need to ensure that the total abnormal rate was at least 3.4%. However, under this circumstance, the threshold is only 78% specific, which means that there are individual CTs whose abnormal rate is less than this, and yet they were still able to achieve an overall sensitivity of 90%. When the laboratory had a sensitivity of 95%, there were no CTs whose screening sensitivity was less than 90%. The specificities of the thresholds were the same as those seen when the laboratory had a screening sensitivity of 88%.

**Table 4:** Thresholds for different surrogates (with laboratory sensitivity 85%, 11 CTs)

	*Threshold sensitivity 85%*	*Sensitivity*	*Specificity*	*Threshold sensitivity 90%*	*Sensitivity*	*Specificity*
Abnormal rate	2.3	100	100	3.4	100	78
ASC rate only	1.3	100	100	1.5	100	100
ASC and ASC-H rate only	1.4	100	100	1.7	100	100
ASC/SIL	1.6	100	78	1.7	100	100

**Table 5:** Thresholds for different surrogates (with laboratory sensitivity 88%, 15 CTs)

	*Threshold sensitivity 85%*	*Sensitivity*	*Specificity*	*Threshold sensitivity 90%*	*Sensitivity*	*Specificity*
Abnormal rate	2.7	100	75	3.1	100	75
ASC rate only	1.2	100	100	1.4	100	67
ASC and ASC-H rate only	1.4	100	75	1.7	100	67
ASC/SIL	1.0	100	87.5	1.0	100	75

## DISCUSSION

The current study is one of the few studies to compare multiple quality measures from gynecologic cytology with actual screening sensitivity to see if they may serve as “surrogates” for true sensitivity. The data we present show that many of these other quality measures may correlate with the true sensitivity of the laboratory, but there are substantial limitations to their use.

By definition, these surrogates are inferior to directly measuring performance because they depend not only on screening sensitivity but on other factors as well.[16] Individual CTs with the same screening sensitivity may have very different thresholds for ASC or LSIL, and this would result in different ASC rates and ASC/SIL ratios and would reduce the correlation with sensitivity.

Interestingly, the correlation of these other quality measures with sensitivity also depends on the overall sensitivity of the laboratory. If the laboratory is performing poorly, then these surrogates perform well. In this setting, there are a lot of mistakes being made, and thus their effect on these surrogates is great. In contrast, when the laboratory is performing well there are few mistakes being made, and the effect of these mistakes on the surrogates is smaller.

The value of these other quality measures to serve as surrogates for sensitivity may also depend on the disease prevalence. In our study the disease prevalence was relatively stable, so we were unable to directly measure the impact of disease prevalence in this study. Further studies may be of value in defining the effect of this variable.

Nevertheless, despite these limitations, minimum standards could be defined that would identify individual CTs who were performing suboptimally. These minimum standards can be identified in laboratories performing at a range of overall sensitivity, and remain both sensitive and specific. Unfortunately, the minimum standards we have identified in this study are often less than one standard deviation away from the mean. This suggests that in order to be most effective, the variation in screening “style” between CTs would need to be substantially reduced. For example, in this laboratory, the ASC/SIL ratio varied from 0.9 to 4.5, a 600% variation. The minimum standards we defined would be much more useful if the variation in the ASC/SIL ratio in the laboratory was less than this. Strategies that improve the precision of these surrogates, such as location-guided screening,[17] may be of value in this setting. While the specific data we present in this report are specific to this laboratory, they suggest that most laboratory's minimum standards would be less than one standard deviation below the mean of the value they are examining.

Interestingly, at all levels of sensitivity studied here, the variation in the abnormal rate or the ASC rate was less than the variation in the ASC/SIL ratio. This is surprising. The ASC/SIL ratio was originally developed because it was thought to vary less than the ASC or total abnormal rate between laboratories.[18,19] Survey data from a variety of laboratories have also noted this trend.[17] Further evaluation may be warranted.

We conclude that the correlation of other quality measures in the gynecologic cytology laboratory with screening sensitivity depends on the overall sensitivity in the laboratory. Thresholds to define minimum screening sensitivity can be defined, but these standards are relatively narrow, and may limit the utility of these standards to serve as surrogates for true screening sensitivity.

## COMPETING INTEREST STATEMENT BY ALL AUTHORS

No competing interest to declare by any of the authors.

## AUTHORSHIP STATEMENT BY ALL AUTHORS

All authors of this article declare that we qualify for authorship as defined by ICMJE http://www.icmje.org/#author.

Each author has participated sufficiently in the work and take public responsibility for appropriate portions of the content of this article.

Each author acknowledges that this final version was read and approved.

## ETHICS STATEMENT BY ALL AUTHORS

This study was conducted with approval from Institutional Review Board (IRB) (or its equivalent) of all the institutions associated with this study. Authors take responsibility to maintain relevant documentation in this respect.
